# Support among healthcare workers for the new mandatory seasonal influenza vaccination policy and its effects on vaccination coverage

**DOI:** 10.1080/07853890.2021.1889022

**Published:** 2021-02-22

**Authors:** Aleksi Hämäläinen, Riitta-Liisa Patovirta, Ella Mauranen, Sari Hämäläinen, Irma Koivula

**Affiliations:** a Institute of Clinical Medicine, University of Eastern Finland, Kuopio, Finland; b Department of Medicine, Kuopio University Hospital, Kuopio, Finland

**Keywords:** Influenza, influenza vaccination, influenza vaccination policy, mandatory vaccination, healthcare workers, act of communicable diseases

## Abstract

**Introduction:**

Finland was the first European country to introduce a nation-wide mandatory seasonal influenza vaccination policy for healthcare workers (HCWs) by mandating that administrators of health care institutions only employ vaccinated HCWs. In this study, we examine the effects of the new policy and the view of HCWs on the new policy.

**Methods:**

A cross-sectional observational study was conducted in Kuopio University Hospital among HCWs working in close patient contact. The statistics on vaccination coverage were obtained from the hospital’s own databases, where employees were asked to self-report their suitability for work. An anonymous survey was sent to HCWs in 2015–2016 (*n* = 987) and 2018–2019 (*n* = 821).

**Results:**

Vaccination coverage increased from 59.5 to 99.6%, according to the hospital’s own records. Among the survey respondents, the seasonal influenza vaccination coverage of HCWs increased from 68.2 to 95.4%. 83.8% of doctors and 49.4% of nurses supported the new policy. 12.7% of doctors and 41.5% of nurses found the new mandate coercive or that it restricted their self-determination.

**Conclusions:**

Our study confirms the positive effects of mandating the administrators of health care institutions to only employ vaccinated HCWs. The majority (57.9%) of all HCWs supported the new policy, with doctors being more compliant than nurses.

**Key messages**
- Finland became the first European country to mandate influenza vaccination for HCWs by mandating that administrators of health care institutions only employ vaccinated HCWs.
- After the new act, the vaccination coverage of HCWs increased close to 100%.
- Most of the HCWs supported the new act and did not find it coercive.

## Introduction

The risk of influenza infection is greater among healthcare workers (HCWs) compared to adults working in non-healthcare settings [1]. Seasonal influenza vaccination is the most effective way to prevent influenza [2]. Among HCWs, vaccination reduces their laboratory-confirmed influenza cases as well as mortality and the prevalence of influenza-like illness in patients of long-term care healthcare facilities [3,4].

Seasonal influenza vaccination coverage has remained low in many European countries despite seasonal vaccination being recommended [5]. The most effective method to increase vaccination coverage among HCWs has been through a mandatory influenza vaccination policy, which has increased vaccination coverage close to 100% [6,7]. In the United States, seasonal influenza vaccination is mandatory in 18 states, but in most of these states there are many means by which to refuse to take the vaccine [8]. In British Columbia, Canada, HCWs either have to wear a surgical mask or take the seasonal influenza vaccination in order to prevent seasonal influenza [9]. Similar policies have been unsuccessfully tried in many other Canadian provinces [10]. A seasonal influenza vaccination mandate by state or government has also been applied in Saudi Arabia, where vaccination is mandatory among certain areas to prevent influenza spreading among pilgrims [11]. In Europe, seasonal influenza vaccination has been mandatory for specific HCW groups in Serbia since December 2017 according to the new rulebook of vaccination, but the law has not yet been implemented. Many other vaccinations are also mandatory for health care personnel in many European countries, including measles–mumps–rubella, tetanus, diphtheria, pertussis, poliomyelitis, hepatitis A, hepatitis B, meningococcus and tuberculosis [12]. There are also mandatory policies for mumps and rubella vaccine, tuberculosis screening and hepatitis B vaccine in many institutions in the United States [13]. Mandatory seasonal influenza vaccination policy among HCWs has decreased nosocomial influenza infections in immunocompromised cancer patients after HCWs increased vaccination rates [14].

In recent studies in the United States, 53.2–74.4% of the surveyed HCWs reported supporting a mandatory influenza vaccination policy [15–18]. Vaccinated HCWs and HCWs working in patient contact have been more supportive towards mandatory vaccination policy than unvaccinated HCWs and HCWs not working in patient contact [15]. There has been no difference in mandatory policy acceptance between doctors, nurses and other hospital employees [17,18]. HCWs who both support and oppose the mandatory vaccination policy have received the same knowledge about influenza and the influenza vaccine, and the vaccination status itself has been a more significant factor in correlating with true and false beliefs of influenza vaccination than supporting or opposing mandatory policy [19]. Regardless of the support towards a mandatory vaccination policy, the majority of HCWs perceived a mandatory vaccination policy as being coercive [16].

Finland became the first European country to mandate administrators of health care institutions to only employ vaccinated HCWs. The New Act of Communicable Diseases was presented on 1 March 2017 and implemented one year later, leaving healthcare administrators some transition time. “For work in client and patient facilities of social welfare and health care units, which are used for treating clients or patients who, based on medical assessments, are susceptible to severe consequences from communicable diseases, a person with inadequate protection from vaccination may only be used in exceptional circumstances. Employees and students in practical training must be protected against measles and varicella, either through vaccination or by having had the disease. In addition, vaccination against influenza is required, as is vaccination against whooping cough for persons treating infants” [20].

Healthcare organizations define the high-risk patients and risk areas by themselves. According to the Finnish institute for health and welfare, high-risk patients include patients with immunodeficiency, children under 1 year of age, elderly persons over 65 years of age and pregnant women. Vaccination is not required for staff working without patient contact or when HCWs only meet patients belonging in risk groups irregularly or only for short periods at a time. Vaccination is required for technicians, physiotherapists, ward clerks, cleaners and other assisting staff with the same preconditions. Many organizations have determined that risk patients are treated in almost all units of their facilities. HCWs working in patient contact face risks treating high-risk patients; therefore, all doctors and nurses working in areas where patients are being treated or transferred must primarily be vaccinated against seasonal influenza. Vaccination is also required for workers who clean, deliver food and manage equipment and for other assisting staff in facilities where patients are being treated or transferred. If an employee refuses to take the seasonal influenza vaccine, they will be primarily transferred to another unit or position with no patient contact. This makes it challenging to strictly comply with these instructions and has led to conflicts between administrators and employees. The new act has sparked debate in the Finnish media.

The aim of the study was to examine the effects of the new, nation-wide policy for HCWs seasonal influenza vaccination and approval of the new policy by the HCWs working in close patient contact.

## Materials and methods

### Data collection

This cross-sectional observational study was carried out in Kuopio University Hospital (KUH). KUH is one of Finland’s five university hospitals. The hospital is a 700-bed teaching hospital that provides tertiary care services to approximately 860,000 citizens in Central and Eastern Finland.

On 31 December 2015, the HCWs of KUH consisted of 728 doctors and 2959 nurses and on 31 December 2018 of 769 doctors and 3009 nurses. The statistics on vaccination coverage were obtained from the hospital’s own databases, where employees were asked to self-report their suitability for work. The database is used by supervisors to verify an employee’s suitability for work and it covers all employees working in patient contact.

The first additional survey was sent in September 2015 before the 2015–2016 influenza season and the second in December 2018 at the beginning of the 2018–2019 influenza season. These voluntary and anonymous surveys were targeted at doctors and nurses who worked in patient contact. Both surveys were created using the Surveypal program and sent to HCWs *via* email together with a cover letter. A notification email and link to the survey was sent to HCWs 1 week after the first email.

### Survey items

The first survey gathered information on profession (doctor, nurse) and whether the respondent had taken or would take the seasonal influenza vaccine during the current influenza season. The second survey gathered information on profession (doctor, nurse), information on previous and current seasonal influenza vaccination status, opinion about the influenza’s severity and views regarding the new Communicable Diseases Act. In both surveys, respondents could choose to leave any of the questions unanswered. Current seasonal influenza vaccination status was asked with the following question “Are you going to, or have you taken the influenza vaccination for the coming flu season?” with the answer options of “Yes”, “No” and “Uncertain”. Previous vaccination status was asked with the question “Have you taken seasonal influenza vaccination during previous years?” with the answer options of “Always”, “Sometimes” and “Never”. Opinion about influenza’s severity was asked with the question “Do you consider influenza as a serious disease?” with the answer options of “Yes”, “No” and “Uncertain”. Support for the new act was asked with the question “Do you support the change in the new Communicable Diseases Act?” with the answer options of “Yes”, “No” and “Uncertain”. Coerciveness of the new act was asked with the question “Do you find the new Communicable Diseases Act as coercive or restricting your self-determination?” with the answer options of “Yes”, “No” and “Uncertain”.

### Data analysis

Data was converted from Surveypal program to a SPSS file, and data analysis was completed using SPSS version 25 (IBM Corp. Released 2017. IBM SPSS Statistics for Windows, Version 25.0. Armonk, NY: IBM Corp.). Statistical analyses used the Chi-square (χ^2^) test for comparison between categorical variables. Fisher’s exact test was used instead of chi-square test to analyze categorical variables, if any cells had low (<5) minimum expected count. In questions with more than two possible answers, the variable analysed was compared to other groups combined. Results with *p*-value lower than 0.05 were counted statistically significant.

### Ethical approval

This study was conducted according to the principles expressed in the Declaration of Helsinki [21] and approved by local ethics committee (the Research Ethics Committee of the Northern Savo Hospital District; 1107/13.02.00/2018).

## Results

According to the hospital’s own database, the official vaccination percentages were 59.5% in the 2015–2016 and 99.6% in the 2018–2019 influenza seasons among all employees working in patient contact.

Altogether, 987 HCWs responded to the additional Surveypal questionnaire in the 2015-2016 survey and 821 in the 2018–2019 survey. In 2015–2016, 202 (20.5%) of the respondents were doctors and 785 (79.5%) were nurses, and in 2018–2019 200 (24.4%) of the respondents were doctors and 621 (75.6%) were nurses. In the first and second surveys, the response rates were 27.7% vs 26.3% among doctors and 26.5 vs. 20.6% among nurses.

The intentions of employees to take the vaccination are presented in Table 1. In 2015–2016, 68.2% of the respondents had taken or were going to take the vaccination. After the new policy in 2018–2019, 95.4% of the respondents had taken or were going to take the vaccination. The change was exceptionally significant among nurses: 62.3 vs. 94.3%. Before the new policy, the intentions of nurses to take the vaccination (62.3%) were significantly (*p* < .001) lower than doctors (91.1%). Subsequently, the difference decreased markedly (doctors 99.0 vs. nurses 94.3%), while still being statistically significant (*p* = .006).

**Table 1.** Answers to the question “Are you going to or have you taken the influenza vaccine in the coming flu season?” and comparison between the years in 2015–2016 (*n* = 987) and in 2018–2019 (*n* = 811).

	2015–2016 *n* (% in the category) *n* = 987	2018–2019 *n* (% in the category) *n* = 811	*p* Value
Will take or have taken the vaccine	673 (68.2)	774 (95.4)	<.001
Doctors	184 (91.1)	196 (99.0)	<.001
Nurses	489 (62.3)	578 (94.3)	<.001
Will not take and have not taken the vaccine	305 (31.9)	22 (2.7)	<.001
Doctors	16 (7.9)	1 (0.5)	<.001
Nurses	289 (36.8)	21 (3.4)	<.001
Uncertain	9 (0.9)	15 (1.8)	.077
Doctors	2 (1.0)	1 (0.5)	.508*
Nurses	7 (0.9)	14 (2.3)	.008

**Table 2.** Previous vaccination status, opinions about influenza and new Act of Communicable Diseases by profession in the 2018–2019 survey.

	All respondents *n* (% in the category)	Doctors *n* (% in the category)	Nurses *n* (% in the category)	*p* Value
Have you taken the seasonal influenza vaccination in previous years?	*n* = 803	*n* = 197	*n* = 606	
Always	525 (64.5)	170 (86.3)	355 (58.6)	<.001
Sometimes	238 (29.6)	22 (11.2)	216 (35.6)	<.001
Never	40 (5.0)	5 (2.5)	35 (5.8)	.070
Do you consider influenza to be a serious disease?	*n* = 814	*n* = 196	*n* = 618	
Yes	729 (89.6)	188 (95.9)	541 (87.5)	.001
No	52 (6.4)	6 (3.1)	46 (8.5)	.029
Uncertain	33 (4.1)	2 (1.0)	31 (5.1)	.013
Do you support the change in the new communicable diseases act?	*n* = 800	*n* = 197	*n* = 603	
Yes	463 (57.9)	165 (83.8)	298 (49.4)	<.001
No	152 (19.0)	15 (7.6)	137 (22.7)	<.001
Uncertain	185 (23.1)	17 (8.6)	168 (27.9)	<.001
Do you find the new communicable diseases act to be coercive or restrictive of your self-determination?	*n* = 812	*n* = 197	*n* = 615	
Yes	280 (34.5)	25 (12.7)	255 (41.5)	<.001
No	448 (55.2)	163 (82.7)	285 (46.3)	<.001
Uncertain	84 (10.3)	9 (4.6)	75 (12.2)	.002

**Table 3.** Support for and opposition to the mandatory vaccination policy (“Yes” and “No” answers in the question “Do you support the change in the new Communicable Diseases Act?”) compared to other categories in the 2018–2019 survey. Uncertain answers removed from all categories.

	Supporting the new act *n* (% in the category)	Opposing the new act *n* (% in the category)	*p* Value
Are you going to or have you taken the influenza vaccine for the coming flu season	*n* = 457	*n* = 140	
Yes	454 (99.3)	124 (88.6)	<.001
No	3 (0.7)	16 (11.4)	
Have you taken seasonal influenza vaccination in previous years?	*n* = 454	*n* = 149	
Always	377 (83.0)	40 (26.8)	<.001
Sometimes or never	77 (17.0)	109 (73.2)	
Do you consider influenza to be a serious disease	*n* = 453	*n* = 138	
Yes	439 (96.9)	110 (79.7)	<.001
No	14 (3.1)	28 (20.3)	
Do you find the new Communicable Diseases Act to be coercive or restrictive of your self-determination?	*n* = 424	*n* = 149	
Yes	38 (9.0)	147 (98.7)	<.001
No	386 (91.0)	2 (1.3)	

*Calculated with Fischer’s Exact Test.

Previous vaccination status, opinion on the influenza’s severity and views towards the new act are shown in Table 2. Most of the respondents had regularly taken the seasonal influenza vaccination in previous years. Doctors had taken the vaccination more often than nurses, and doctors considered influenza as a serious illness more often than nurses. Merely half of the nurses (49.4%) and the majority of doctors (83.8%) supported the new act. Only a few doctors (12.7%) reported finding the new act coercive, compared to 41.5% of nurses.

In addition to the profession, other factors and beliefs behind support for and opposition to the new mandatory vaccination policy are presented in Table 3. 99.3% of the HCWs supporting and 88.6% of the HCWs opposing the new mandatory vaccination had taken or were going to take the seasonal influenza vaccination in the coming flu season. HCWs supporting the new act had taken seasonal influenza vaccination more often (83.0%) in previous years than HCWs who opposed the new act (26.8%). 79.7% of the HCWs opposing the new act considered influenza to be a serious disease, compared to 96.9% of the HCWs supporting the new act. 91.0% of the HCWs supporting the new act did not find the new act coercive or restrictive of self-determination, whereas 98.7% of the respondents opposing the act found the new act coercive or restrictive of self-determination.

## Discussion

In this study, we examined the effects of the nation-wide new policy for the seasonal influenza vaccination of HCWs and the approval of the new policy by HCWs working in close patient contact in a Finnish tertiary care hospital. The new Finnish policy to only employ vaccinated HCWs in patient facilities where patients are susceptible to severe consequences from influenza is the world’s first nation-wide policy to increase the vaccination coverage of HCWs by mandating administrators of health care institutions by law instead of mandating HCWs to take the vaccine.

After implementation, the vaccination coverage of HCWs in the study hospital increased from 59.5 to 99.6% according to the hospital’s own statistics, where all employees with patient contact were asked to inform their supervisors of their suitability for work. Similar results were found from anonymous survey reports: vaccine coverage increased from 68.2 to 95.4%. The increase in vaccination coverage is in accordance with other similar studies [7]. In particular, the vaccination coverage of nurses increased markedly. However, the increase in the vaccination coverage of doctors was also statistically significant.

The majority (57.9%) of the respondents supported the new seasonal influenza vaccination policy. Support towards the mandatory policy among all employees is similar to that of previous studies [15–18]. Doctors were more supportive and did not find the new act to be as coercive as nurses. The difference was remarkable without a definite explanation. In our study, doctors supported mandatory vaccination policy significantly more (83.8%) than in previous studies (56.2–65.3%), whereas the support among nurses towards the mandatory vaccination policy (49.4%) was close to results from previous studies (51.1–55.9%) [17,18]. Vaccinated HCWs were more supportive towards the mandatory vaccination policy in our study (99.3%) compared to an earlier study (79%) from the United States [15]. 88.6% of the HCWs opposing the new mandatory policy were going to take or had taken the seasonal influenza vaccination for the coming flu season, referring to the fact that the majority of the HCWs opposing the act will take the seasonal influenza vaccine despite their opposition. HCWs supporting the new act considered influenza more often as a serious disease than HCWs who opposed the new act, but the majority of HCWs opposing the new act still considered influenza to be a serious disease. Douville et al. [19] have suggested that employees who oppose mandated vaccination seem to fall into two groups: one group who misunderstand the facts about influenza and the second group who understand the facts but disagree with the value thinking that a decision regarding immunizations should be individual. Nearly all (98.7%) of the HCWs who oppose the new act in our study found the new act to be coercive or self-restricting. The findings in our study suggest that nearly all HCWs who oppose the new act believe that an immunization decision should be individual and a person’s individual choices, whereas a minority of the HCWs who oppose the mandate do not consider influenza to be a serious enough disease to take the vaccination.

The number of “uncertain” answers in our study was also high in the question regarding support for the new act. In the study hospital, each department was asked to independently evaluate facilities where patients, as a rule, are susceptible to severe consequences for communicable diseases. In these facilities, vaccinating the entire staff was recommended. Therefore, different units in the same hospital might have had different policies demanding the seasonal influenza vaccination and explaining the high percentage of “uncertain” in the answers.

This study has several limitations. First, this study does not represent Finland nationwide, rather only a single tertiary care hospital. The survey was completed anonymously in order to gather reliable data. While the survey response rates were not higher than 21–27% among almost 4,000 employees, they were enough for a quantitative study. Second, in the surveys, we used the intentions of employees to get vaccinated as the actual vaccination percentage in order to increase the survey response rates, because the debate regarding the seasonal influenza vaccination and the intention of HCWs to get vaccinated are most intense in the autumn at the beginning of the vaccination season. In one other study, the intention to get vaccinated predicted 58% of a vaccination uptake among health care professionals [22]. Third, according to Finnish law, employers are not allowed to access employees’ medical records without permission. Therefore, data from hospital’s own databases is based on self-reporting and cannot be confirmed from employees’ medical records. Nevertheless, the results from the hospital’s own database and our anonymous survey converge, indicating that the increase in vaccine coverage is reliable.

Mandating healthcare institutes to only employ vaccinated HCWs instead of mandating HCWs to take the vaccine seems to be an equally good way to increase seasonal influenza vaccination coverage as mandatory seasonal influenza vaccination for employees. After implementing the new policy, recent announcements from other Finnish university hospitals also show very high 90–95% seasonal influenza vaccination coverage among HCWs [23,24]. In the study hospital, the coverage rose from 59.5 to 99.6%. In one study from the United States, high vaccination rates after mandating seasonal influenza vaccination for HCWs have sustained over several years [25]. We hope that results in Finnish policy will last similarly in the coming years and that, in time, HCWs will accept the seasonal influenza vaccination as naturally as other patient safety policies without any mandatory actions.

## Data Availability

Due to the nature of this research, the participants in this study did not agree for their data to be shared publicly, so supporting data is not available.
